# Genotoxic Effects of Etoposide, Bleomycin, and Ethyl Methanesulfonate on Cultured CHO Cells: Analysis by GC-MS/MS and Comet Assay

**DOI:** 10.1155/2020/8810105

**Published:** 2020-07-30

**Authors:** Donald H. Atha, Erdem Coskun, Onur Erdem, Alessandro Tona, Vytas Reipa, Bryant C. Nelson

**Affiliations:** ^1^National Institute of Standards and Technology, Biosystems and Biomaterials Division, Materials Measurement Laboratory, Gaithersburg, MD 20899, USA; ^2^National Institute of Standards and Technology, Biomolecular Measurement Division, Materials Measurement Laboratory, Gaithersburg, MD 20899, USA; ^3^University of Maryland, Institute for Bioscience and Biotechnology Research, Rockville, MD 20850, USA; ^4^University of Health Sciences Turkey, Department of Pharmaceutical Toxicology, Gulhane Faculty of Pharmacy, 06010 Ankara, Turkey

## Abstract

To evaluate methods for analysis of genotoxic effects on mammalian cell lines, we tested the effect of three common genotoxic agents on Chinese hamster ovary (CHO) cells by single-cell gel electrophoresis (comet assay) and gas chromatography-tandem mass spectrometry (GC-MS/MS). Suspension-grown CHO cells were separately incubated with etoposide, bleomycin, and ethyl methanesulfonate and analyzed by an alkaline comet assay and GC-MS/MS. Although DNA strand breaks were detected by the comet assay after treatment with all three agents, GC-MS/MS could only detect DNA nucleobase lesions oxidatively induced by bleomycin. This demonstrates that although GC-MS/MS has limitations in detection of genotoxic effects, it can be used for selected chemical genotoxins that contribute to oxidizing processes. The comet assay, used in combination with GC-MS/MS, can be a more useful approach to screen a wide range of chemical genotoxins as well as to monitor other DNA-damaging factors.

## 1. Introduction

Exposure of mammalian cells to genotoxic agents and ionizing radiation can result in a wide range of adverse effects, including lipid, protein, and DNA damage. DNA damage is implicated in the development of mutagenesis, carcinogenesis, and pathogenesis of several diseases including, Huntington's, Parkinson's, Alzheimer's, and acquired immune deficiency syndrome [1]. Ionizing radiation is another DNA damage source, which is also widely used in clinical practice for cancer treatment [2]. Experimental in vitro evidence led to the hypothesis that complex damage that includes both strand breaks and base lesions is more difficult to repair by repair enzymes [3]. In this regard, it is critical to have reliable methods to assess both base lesions and strand breaks as induced by chemical agents by detecting the resulting damage to cellular DNA. For this, we compared gas chromatography-tandem mass spectrometry (GC-MS/MS) and the comet assay in detecting DNA damage from three commonly used genotoxic agents: etoposide, bleomycin, and ethyl methanesulfonate (EMS), which are known to have different mechanisms of action and levels of toxicity.

Etoposide is a podophyllotoxin glycoside with a D-glucose derivative that forms a ternary complex with DNA and topoisomerase II, prevents religation of the DNA strands, and causes DNA strands to break. Cancer cells are more affected than normal cells, since cancer cells divide faster. Treatment with the drug results in errors in DNA synthesis which promote apoptosis of the cancer cells [4–6].

Bleomycin sulfate is a glycopeptide antitumor antibiotic. It induces DNA strand breaks, which *in vitro* depend on oxygen and metal ion concentrations. Its mechanism of action is not completely understood, but it is thought that bleomycin chelates metal ions (primarily iron) and forms a pseudoenzyme, which reacts with oxygen to produce superoxide and hydroxyl radicals, which can induce base lesions and strand breaks [7–9].

Ethyl methanesulfonate (EMS) is a potentially carcinogenic compound that causes point mutations in DNA by nucleotide substitution. The ethyl group of EMS reacts with guanine bases by alkylation, forming *O*^6^-ethylguanine. DNA polymerases then substitute thymine in place of cytosine opposite the *O*^6^-ethylguanine. As a result, during replication, the original G:C base pair becomes an A:T mutation [10, 11].

Several assays exist for the analysis of damage to genomic DNA. These include measurement of DNA damage in whole cells using the single-cell gel electrophoresis (comet assay—alkaline and neutral), the micronucleus assay, and the measurement of fragmented DNA in isolated DNA using capillary or gel electrophoresis. Both gas chromatography-mass spectrometry (GC-MS) and liquid chromatography-mass spectrometry (LC-MS), in single or tandem versions, have been used for the measurement of numerous oxidatively modified DNA products such as sugar and base lesions, 8,5′-cyclopurine-2′-deoxynucleosides, base-base tandem lesions, and DNA-DNA and DNA-protein crosslinks, *in vitro* and *in vivo*. The comet assay is a popular method that is used to detect clinically relevant levels of DNA damage in the form of strand breaks and alkali-labile sites [12–14]. Mass spectrometry (MS) provides structural evidence for an analyte and enables accurate quantification by using stable isotope-labeled analogs of analytes as internal standards (MS with isotope dilution).

In this study, we compare the comet assay and GC-MS/MS in their ability to detect structural changes in the DNA of cultured mammalian cells after treatment with three common chemical genotoxins: etoposide, bleomycin, and EMS. These agents were chosen since they are all known to induce DNA damage but by different mechanisms of action and toxicity. In this regard, we chose specific conditions of exposure for each agent that was known to produce significant genotoxicity with minimal loss in cell viability [15–17]. Chinese hamster ovary (CHO) cells were exposed to the three agents during suspension culture, and the extent of DNA damage was measured by both analytical techniques on identical sample sets. Strand breaks were quantified via the comet assay, and the identities and levels of induced DNA base lesions were quantified by mass GC-MS/MS. We used the alkaline comet assay rather than the neutral comet assay since the alkaline version is effective at detecting both single- and double-strand breaks. Although the FPG-modified comet has been shown to be useful in detecting oxidized bases, we chose GC-MS/MS for this purpose in that it provides a direct chemical analysis of the structure and quantity of several oxidation products. In this regard, we wanted to use the alkaline comet assay to specifically compare the measurement of DNA damage in the form of strand breakage (single and double) with GC-MS/MS to detect base lesions. By comparing the effectiveness of the alkaline comet assay to GC-MS/MS, we show that paired measurements of DNA strand breaks and DNA lesions, using these two methods, provide insight into the proportions of these types of damage produced by toxins with a widely varying mechanism of action and potentially by ionizing radiation.

## 2. Materials and Methods

### 2.1. Materials

Freestyle suspension, Chinese hamster ovary (CHO-S) cells were obtained from Invitrogen (Carlsbad, CA, USA). Freestyle CHO expression medium was obtained from Invitrogen (Cat. No. 12651) and supplemented with L-glutamine to a final concentration of 8 mmol/L before use. Etoposide was obtained from Sigma-Aldrich Corp. (Cat. No. E1383-25mg, St. Louis, MO, USA). Bleomycin sulfate (Cat. No. B8416) and ethyl methanesulfonate (EMS, Cat. No. M0880-1g) were obtained from Sigma-Aldrich Corp. Calf thymus DNA-sodium salt (ct-DNA), sodium phosphate monobasic, and sodium phosphate dibasic were purchased from Sigma-Aldrich Corp. 4,6-Diamino-5-formamidopyrimidine-[^13^C,^15^N_2_] (FapyAde-^13^C,^15^N_2_), 2,6-diamino-4-hydroxy-5-formamidopyrimidine-[^13^C,^15^N_2_] (FapyGua-^13^C,^15^N_2_), 8-hydroxyadenine-[^15^N_5_] (8-OH-Ade-^13^C,^15^N_2_), 5-hydroxy-5-methylhydantoin-[^13^C,^15^N_2_] (5-OH-5-MeHyd-^13^C,^15^N_2_), and 8-hydroxy-2′-deoxyguanosine-[^15^N_5_] (8-OH-dGuo-^15^N_5_), also known as 8-oxo-7,8-dihydro-2′-deoxyguanosine-[^15^N_5_], were purchased from Cambridge Isotope Laboratories (Andover, MA). 8-Hydroxyguanine-[^15^N_5_] (8-OH-Gua-^15^N_5_), also known as 8-oxo-7,8-dihydroguanine-[^15^N_5_] (8-oxoGua-^15^N_5_), was obtained by hydrolysis of 8-hydroxy-2′-deoxyguanosine-[^15^N_5_] with 60% (*v*/*v*) formic acid at 140°C for 30 min followed by lyophilization. Subsequently, 8-OH-Gua-^15^N_5_ was dissolved in 10 mmol/L NaOH before use. *Escherichia coli* formamidopyrimdine DNA glycosylase (Fpg) and *Escherichia coli* endonuclease (III) (EndoIII) were purchased from Trevigen (Gaithersburg, MD, USA).

### 2.2. Cell Culture of Freestyle CHO-S Cells

Freestyle suspension, CHO-S cells were grown up to 1 × 10^6^ cells/mL in 250 mL spinner flasks (Corning, Cat. No. 4500-250) at 37°C, 90% relative humidity, and 8% CO_2_. Cell counts for viability were performed in duplicate after each treatment using the Beckman/Coulter Vi-cell XR cell counter. All cellular viability counts showed readings 98.1% or above except for one preparation of EMS at 800 *μ*g/L, which was 96%.

### 2.3. Etoposide Treatment

Four spinner flasks containing 100 mL each of cell suspension (1 × 10^6^ cells/mL) were prepared. Etoposide stock solution (10 mg/mL) was prepared in dimethylsulfoxide (DMSO). Cells were exposed at [0, 1.5, 3, and 6] *μ*g/mL for 1 h on an orbital shaker at 37°C, 90% relative humidity, and 8% CO_2_ as follows: at time 0, 60 *μ*L of DMSO was added to control flask #1, 15 *μ*L of stock etoposide solution+45 *μ*L of DMSO was added to flask #2, 30 *μ*L of stock solution+30 *μ*L of DMSO was added to flask #3, and 60 *μ*L of stock solution was added to flask #4. After 1 h, flasks were removed from the incubator and placed on ice. Aliquots for Vi-cell count (viability) checks were removed from each flask. The remaining suspensions were transferred to 50 mL tubes and spun down at 4°C. The cells were suspended in cold medium and placed on ice while performing Vi-cell analysis (15 min.). Cells were spun down again, supernatant was discarded, and cells were again resuspended in freezing medium (CHO expression medium+10% DMSO). 1 mL aliquots at a cell density of 6 × 10^6^ cells/mL were prepared, quickly frozen, and stored at –150°C for the comet assay and GC-MS/MS.

### 2.4. Bleomycin Treatment

Four spinner flasks containing 100 mL each of cell suspension (1 × 10^6^ cells/mL) were set. Bleomycin stock solution (5 mg/mL) was prepared in water. Cells were exposed at [0, 0.5, 1, and 2] *μ*g/mL for 1 h on an orbital shaker at 37°C, 90% relative humidity, and 8% CO_2_ as follows: at time 0, 40 *μ*L of water was added to control flask #1, 10 *μ*L of stock bleomycin solution and 30 *μ*L water were added to flask #2, 20 *μ*L of stock bleomycin stock solution and 20 *μ*L of water were added to flask #3, and 40 *μ*L of stock bleomycin solution was added to flask #4. After 1 h, the flasks were removed from the incubator and placed on ice. For each flask, aliquots for Vi-cell count were removed; remaining suspensions were transferred to 50 mL tubes, spun down, and resuspended in cold medium; and 1 mL aliquots were prepared exactly as described previously for etoposide.

### 2.5. EMS Treatment

Four spinner flasks containing 100 mL each of cell suspension (1 × 10^6^ cells/mL) were prepared. EMS stock solution (1.206 g/mL) was prepared in deionized water. Cells were exposed at [0, 400, 800, and 1,600] *μ*g/mL for 4 h on an orbital shaker at 37°C, 90% relative humidity, and 8% CO_2_, as follows: at time 0, nothing was added to control flask #1, 33.2 *μ*L of EMS stock solution was added to flask #2, 66.3 *μ*L of EMS stock solution was added to flask #3, and 132.7 *μ*L of EMS stock solution was added to flask #4. After 4 h, the flasks were removed from the incubator and placed on ice. For each flask, aliquots for Vi-cell count were removed; the remaining suspension was transferred to 50 mL tubes, spun down, and resuspended twice in cold medium; and 1 mL aliquots were prepared as described previously for etoposide and bleomycin.

### 2.6. Comet Assay

DNA single- and double-strand breaks as well as alkali-labile sites were measured by the alkaline comet assay as described previously [18]. The procedure is similar to that employed in many previous studies [19–22]. Briefly, each well of a 20-well CometSlide (Trevigen, Inc., MD, USA, Cat. No. 4252-200-01) was filled with 30 *μ*L of a thoroughly mixed suspension of cells in low-melting agarose (2 × 10^4^ cells/mL). The slides were set up in triplicate, each using 5 wells at each treatment concentration. After incubating the slides for 30 min in cold (4°C) cell lysis solution (Trevigen, Inc., Cat. No. 4250-010-01) and then 20 min at room temperature (23°C ± 1°C) in alkaline denaturing and unwinding solution (200 mmol/L NaOH, 1 mmol/L EDTA, and pH > 13), electrophoresis was performed at 4°C in a CometAssay ES tank filled with alkaline solution (Trevigen, Inc., MD, USA) for 30 min. at 21 V (1 V/cm). The slides were then rinsed with distilled water, fixed 5 min in 70% ethanol, dried, then stained 5 min at 4°C with SYBR Green I (Trevigen, Inc., Cat No. 4250-050-05), diluted 1 : 10 000 in TE buffer pH 7.5 (10 mmol/L Tris, 1 mmol/L EDTA), drained to remove excess staining solution, and thoroughly dried at room temperature in the dark.

### 2.7. Microscopic Image Analysis

Comet slides were analyzed as described previously in [23]. Briefly, slides were visualized by epifluorescence microscopy using an Olympus System Model BH-2 microscope, equipped with the appropriate optical filter set for SYBR Green I (460 nm excitation and 560 nm emission wavelengths, Chroma, 49002 ET GFP). A LUDL MAC 6000 automated stage and a Photometrics Snapcool HQ2 monochrome CCD camera were controlled using Nikon Elements BR software (ver. 4.20, Nikon Inc.). Integrated intensities and percent DNA in the tail were determined using ImageJ (ver. 1.47v, NIH) and CometScore Pro (ver. 1.01.44, TriTek Corp., VA, USA) software utilizing the following equations:

(1) $$\begin{array}{c} \text{Total head intensity }I_{\text{h}}=\sum I_{\text{h}\left(x,y\right)},\\ \text{Total tail intensity }I_{\text{t}}=\sum I_{\text{t}\left(x,y\right)},\\ \%\text{DNA in tail}=\frac{100I_{\text{t}}}{I_{\text{h}}+I_{\text{t}}}, \end{array}$$

where *I*_h(*x*, *y*)_ and *I*_t(*x*, *y*)_ are the individual pixel intensities within the head and tail regions of the comet image. Approximately 100 cells were counted at each treatment level for each of three replicates.

### 2.8. Isolation of DNA from Treated Cells for GC-MS/MS Analysis

DNA was extracted from treated cells using high-salt extraction exactly as described previously [24]. The method is based on a procedure published earlier [25]. Briefly, the cells were centrifuged and washed twice in phosphate-buffered saline (PBS) and incubated for 18 h at 37°C in freshly prepared lysis solution (pH 8, 10 mmol/L Tris, 20 mmol/L EDTA, 10 mmol/L NaCl, 1% SDS) containing proteinase K (2 mg/mL). After incubation, the sample was placed on ice, and saturated NaCl (6 mol/L) was added, vortexed until cloudy, incubated 10 min at 56°C, and centrifuged to remove precipitated proteins. The supernatant containing DNA was carefully decanted, and incremental amounts of cold 96% ethanol were added to obtain visible DNA and centrifuged. The DNA pellet was washed twice in cold 70% ethanol and centrifuged, and the remaining ethanol evaporated by vacuum desiccation with Drierite. The DNA sample was then dissolved in TE buffer (pH 8.0) containing 0.2 mg/mL RNase A/T1, incubated for 1 h at 37°C, precipitated again in cold 96% ethanol, and collected by spooling into a 1.5 mL tube. The DNA pellet was again washed twice in 70% ethanol, evaporated as before, and dissolved in 0.5 mL deionized water overnight on a shaker maintained at 4°C. The sample DNA concentration (*μ*g/*μ*L) was then measured by absorbance at *λ* = 260 nm on a NanoDrop One and aliquoted as 50 *μ*g for GC-MS/MS analysis.

### 2.9. Measurement of Modified DNA Bases by GC-MS/MS

Quadruplicate samples containing 50 *μ*g each of genomic DNA extracted from etoposide-, bleomycin-, and EMS-treated cells were enzymatically hydrolyzed, derivatized by trimethylsilylation, and analyzed using DNA extraction and isotope dilution GC-MS/MS methodology exhaustively described in previous studies [26–29]. Samples of 40 Gy gamma irradiated DNA from calf thymus were used as positive controls (e.g., identification of modified DNA bases).

The multiple-reaction-monitoring (MRM) mode GC-MS/MS analyses were conducted based on modifications of a previously developed selected-ion-monitoring (SIM) mode GC/MS method [30]. Stable isotope-labeled analogues of modified DNA bases were utilized as internal standards to positively identify and accurately quantify the DNA base lesions. All MS analyses were performed on an Agilent 7000 series triple quadrupole GC-MS/MS system (Agilent Technologies, Santa Clara, CA) operated in positive ion mode with electron ionization. The modular system consisted of a 7693 autosampler, a 7890A GC oven, and a 7000 series triple quadrupole mass analyzer set to the widest resolution for MS1 and MS2. See Supplementary Materials for further detail, mass transitions, etc. Final results are reported in terms of the number of lesions quantified/10^6^ DNA bases.

### 2.10. Statistical Analysis

All statistical analyses including means, standard error, and standard deviations and uncertainties were conducted with GraphPad Prism 6.0. Uncertainties, compared to controls, were calculated using the unpaired one-tailed *t*-test.  Table 1 contains the results for the comet assay analyses, and Table 2 contains the results for the GC-MS/MS analyses.

## 3. Results

### 3.1. Analysis by Comet Assay

**Figure 1:**
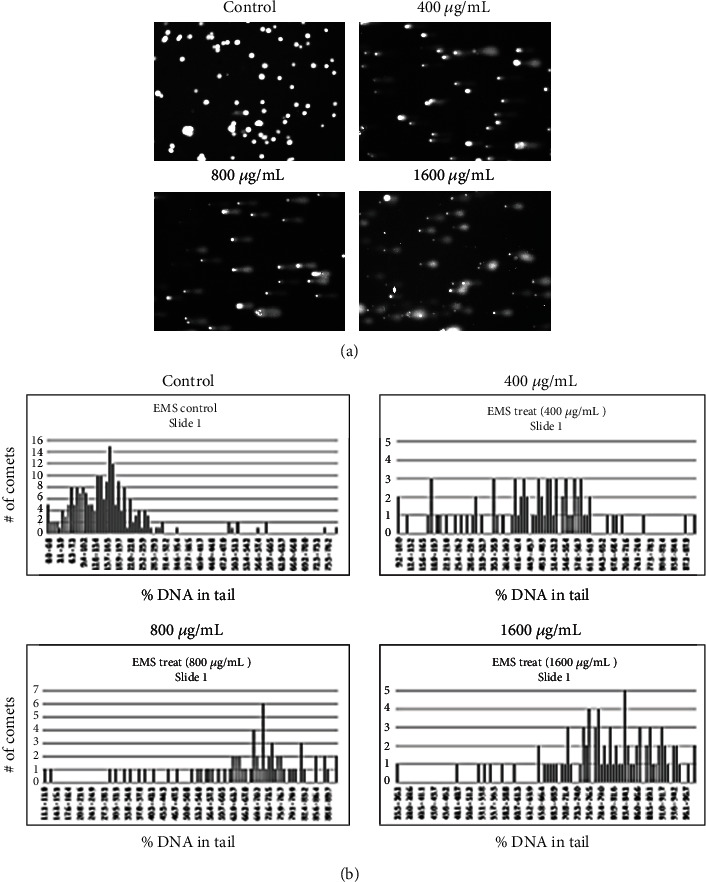
Comet assay measurement of DNA damage. (a) Typical comet assay images of cells after treatment with increasing concentrations of EMS. (b) From multiple images at each treatment level, the % DNA in tail was determined for individual comets and plotted as histograms.

Figure 1(a) shows typical images of comet assay data from cells that were treated with increasing concentrations of EMS. The % DNA in tail was measured at each concentration and plotted as typical histograms (x-axis scale is 0–100% DNA in tail), as shown in Figure 1(b).

**Figure 2:**
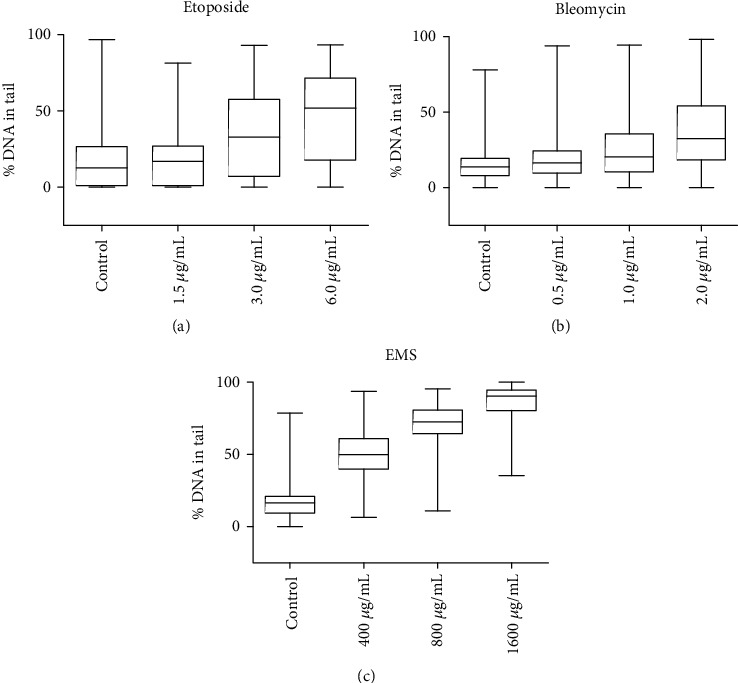
Comet assay—comparison of treatment with (a) etoposide, (b) bleomycin, and (c) EMS. The data are plotted as box and whiskers, which indicates the 25 and 75 percentiles as the size of the data box, the median % DNA as a horizontal line within the boxes, and the outliers by the max and minimum values of the vertical lines. Approximately 100 cells were counted at each treatment level. The data are from one of three replicates.

**Figure 3:**
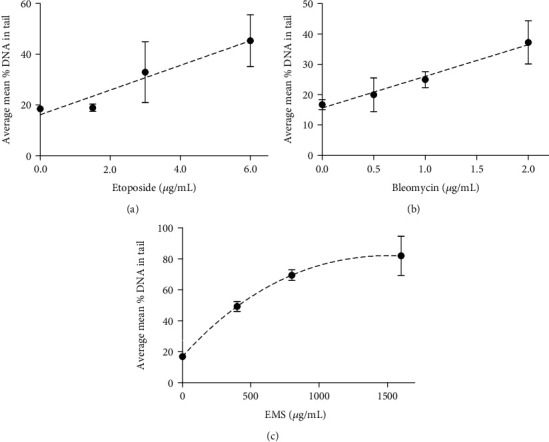
Comet assay—comparison of treatment by (a) etoposide, (b) bleomycin, and (c) EMS. The data are plotted as the average of the means of 3 separate comet assay replicates performed in parallel on the same set of treated samples. Statistical analysis is given in Table 1.

Figure 2 shows the box and whiskers plots of comet analysis in which increase in the % DNA in tail was observed for cells treated with increasing concentrations of etoposide, bleomycin, and EMS. The EMS treatment resulted in substantial DNA damage (80% at the highest treatment level). However, the etoposide and bleomycin data indicated lower damage (30% to 50%) but more heterogeneity in the distribution of comets, which reflected an increased biovariability in responding to these treatments. For this, it was necessary to average three sets of replicate comet data to obtain adequate statistics.  Figure 3 shows plots of the average means of the 3 individual replicate sets of comet assay measurements using the same set of etoposide-, bleomycin-, and EMS-treated cells. The plots indicate the same trends shown in Figure 2.

Table 1 describes the statistical analysis of the 3 separate replicate comet assay measurements, performed in parallel, using the same set of etoposide-, bleomycin-, and EMS-treated cells. The average of the 3 individual means and the standard deviations of 3 means were determined for each treatment level. Although the comet assays were performed on the same day in parallel, using the same set of treated cells, the result is a reflection of the variation between individual comet measurements. The uncertainties (*p* value vs. control) remain high for the etoposide and bleomycin at the lowest concentration but are significantly lower at higher concentrations. The uncertainties for the EMS-treated remain low (*p* < 0.0001) at all concentrations tested.

### 3.2. Analysis by GC-MS/MS

**Figure 4:**
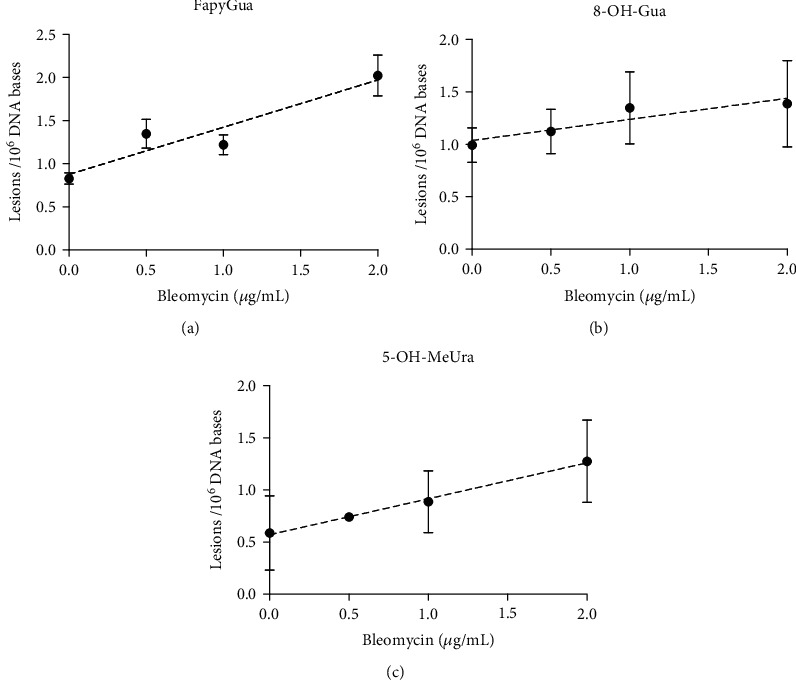
GC-MS/MS—comparison of the induction of (a) FapyGua, (b) 8-OH-Gua, and (c) 5-OH-MeUra by bleomycin. The data are plotted as the mean and standard deviation of 4 separate replicates, performed in parallel, as described in Materials and Methods. Statistical analysis is given in Table 2.

Samples containing 50 *μ*g of genomic DNA extracted from etoposide-, bleomycin-, and EMS-treated cells were enzymatically digested and analyzed using isotope-dilution GC-MS/MS. The following DNA base lesions were detected and quantified: 8-OH-Gua, FapyGua, 5-OH-MeUra, FapyAde, 5-OH-5-MeHyd, 5-OH-Cyt, 5-OH-Ura, and 8-OH-Ade (data shown in Figure S1). Bleomycin is the only agent which produced significant increases in the level of DNA lesions. In Figure S1, the first three lesions (8-OH-Gua, FapyGua, and 5-OH-MeUra) that showed increasing trends with bleomycin are compared to the lack of any significant increases after treatment with etoposide and EMS. The remaining lesions (FapyAde, 5-OH-5-MeHyd, 5-OH-Cyt, 5-OH-Ura, and 8-OH-Ade), which did not show significant increases after treatment with etoposide, bleomycin, and EMS, are also shown in Figure S1. Plots of the means and standard deviations of the three lesions with significant increases (8-OH-Gua, FapyGua, and 5-OH-MeUra), produced by bleomycin, are shown in Figure 4.

As shown in Figure 4, the FapyGua measurements resulted in the least variation and the largest increase relative to the controls. The 8-OH-Gua measurements showed much higher variation and lower increases. The slightly higher number of lesions in the untreated control (1.0/10^6^ DNA bases) may indicate an increase in the induction of 8-OH-Gua lesions during DNA isolation. However, the GC-MS/MS method used here has been shown to be reliable and reproducible for the measurement of 8-OH-Gua levels when compared to an alternative sample preparation procedure combined with LC-MS/MS [31]. An upward trend with concentration was again observed in the 5-OH-MeUra measurements, despite the high variation and low statistical significance (Table 2).

Table 2 shows the statistical analysis of GC-MS/MS measurements after treatment with bleomycin. The table includes mean number of DNA base lesions per million bases, including the standard error and deviation of the mean and the individual uncertainties vs. control (*p* values) as a function of treatment concentration. Although the individual measurements of 8-OH-Gua and 5-OH-MeUra at the lowest treatment concentration had low significance values (*p* = 0.2), compared to control measurements, at the highest treatment concentrations, a higher significance (*p* = 0.06 and *p* = 0.02) was observed. For the bleomycin treatment using the FapyGua lesion, a significance (*p* = 0.001) was obtained.

## 4. Discussion

Since chemical agents are used for clinical use, specifically for their toxic effects on cancer cells, it is important to develop sensitive assays to monitor their effects. In this regard, the comet assay has been used extensively to test the effect of genotoxic agents on DNA strand breakage in mammalian cells. In the current study, we tested the effect of three commonly used genotoxic agents, etoposide, bleomycin, and EMS, which are known to have different mechanisms of action, to determine if DNA damage in the form of strand breaks is correlated with base lesion formation by these agents. So that this comparison would be valid for living cells, we chose treatment conditions for each agent which would result in minimal loss in viability.

We found that the comet assay could detect DNA strand breakage induced by all three of the test agents. EMS yielded a much higher response under the conditions we chose for treatment. The range in concentration chosen for EMS was based on previously published studies that indicated its effectiveness at producing strand breaks [17] which we found producing a loss of cellular viability of less than 2%. However, EMS, which yielded the highest effectiveness in producing strand breaks (approaching 90%), produced no detectable increase in DNA base lesions, despite its high treatment concentration. In this case, there was no observed correlation between the production of strand breaks and nucleobase oxidation by this agent. Perhaps, the use of a more potent alkylating agent or cisplatin would produce nucleobase oxidation detectable by GC-MS/MS. Bleomycin was the only agent that produced detectable base lesions, possibly due to the proposed mechanism which leads to the generation of free radicals.

Topoisomerase II functions to untangle DNA strands by a series of stand break and ligation steps. The absence of lesions induced by etoposide is consistent with what is known of the mechanism of etoposide, which inactivates the ligation function of topoisomerase II without reacting with the DNA bases [4]. This information is in agreement with the lack of increased modified DNA bases detected by GC-MS/MS.

EMS acts to produce mutations by guanine alkylation to form the base lesion O^6^-ethylguanine. However, this particular labeled base lesion could not be included in the present study because the format of our current analytical methodology does not allow for the detection or quantitation of alkylation damage to the DNA bases. The formation of the transition mutation can often lead to DNA strand cleavage as observed in our study. Bleomycin, on the other hand, may chelate metal ions and act as a pseudoenzyme that reacts with oxygen to produce free radicals that cleave DNA and also oxidize bases to produce the increase in lesions we observed [10].

Approximately 10^4^ to 10^6^ DNA lesions are induced in each living mammalian cell every day, primarily due to spontaneous reaction with water and reactive oxygen species (ROS), produced during normal metabolism, by replication errors during DNA synthesis or by exogenous factors such as chemicals or ionizing radiation [32]. If unrepaired, these lesions can result in DNA damage in the form of mutations, double-strand breaks, and ultimately chromosomal rearrangements and translocations leading to cancer and cell death. Single-strand breaks are relatively easy to repair by cellular ligases and therefore are repaired in a matter of minutes (*t*_1/2_ ᵙ 20 min). Double-strand breaks, particularly for nonhomologous end joining, are much more difficult to repair, take much longer, and are more subject to error [33]. This is consistent with the enhanced presence of DNA strand breaks we observed after EMS treatment, as measured by the comet assay.

Oxidatively induced damage to DNA bases can be generated by ROS attack (predominantly hydroxyl radical (^·^OH)) on purine and pyrimidine nucleotides in the nucleotide pool and on intact duplex DNA. Because guanine possesses the lowest oxidation potential [34], oxygen radical attack often results in the incorporation of 8-OH-dGTP into the nucleotide pool along with the simultaneous induction and cellular accumulation of the highly mutagenic lesion, 8-OH-Gua [9, 29]. 8-OH-Gua promotes G → T transversion mutations that can also lead to strand breakage in the process [10]. In this regard, increases in both the DNA base lesion (8-OH-Gua) and DNA strand breakage were observed in our GC-MS/MS and comet assay measurements, respectively, after treatment with bleomycin. Despite the apparent higher sensitivity of the comet assay, in the case of bleomycin, both GC-MS/MS and comet assay are viable methods to detect DNA damage.

Repair of some DNA lesions can be accomplished through base excision repair (BER). Since the overall structure at the affected site is basically intact, this multistep process usually occurs faster and with higher efficiency and fidelity than the repair of double-strand breaks [33, 35]. DNA containing single- and double-strand breaks may not contain a large number of DNA base lesions. This would explain why EMS, which produced a high level of DNA strand breaks, as measured by the comet assay, did not show a significant increase in DNA base lesions, as indicated by GS-MS/MS. In addition, BER may reduce the level of DNA base lesions during treatment. For these reasons, the comet assay would be more sensitive in detecting EMS-induced DNA damage. Treatment with etoposide, on the other hand, also produced an increase in DNA strand breaks under our experimental conditions but with no increase in DNA base lesions. This agent interferes with DNA replication by blocking religation of strand breaks and, unlike bleomycin, does not have the ability to chelate iron, which can catalyze the formation of ROS to produce base lesions. In this regard, paired analysis of both DNA strand breakage and base damage can also be useful after treatment by radiation in assessing biologic harm and the potential for DNA repair [2, 3].

## 5. Conclusions

GC-MS/MS in general is a critical tool in the analysis of genotoxicity. However, under our experimental conditions, it was limited in detecting the effects of chemical genotoxins such as etoposide and EMS. However, for specific agents such as bleomycin, the detection of base lesions by GC-MS/MS may be preferable to obtain accurate quantification. Under our experimental conditions, the comet assay for strand breaks was shown to be effective in detecting DNA damage caused by all three of these agents. Used in combination with GC-MS/MS, the comet assay would be useful to screen a wide range of other chemical genotoxins, such as cisplatin, and to provide insight into mechanisms of complex DNA damage.

## Figures and Tables

**Table 1:** Statistical analysis of multiple comet assays (% DNA in tail).

Etoposide	Control	1.5 *μ*g/mL	3.0 *μ*g/mL	6.0 *μ*g/mL
Mean (Av. of 3 means)	18.46	18.86	32.92	45.34
Stand Dev., *n* = 3	0.894	1.190	9.807	8.301
Stnd. error of mean	0.6324	0.8416	6.934	5.870
*p* value (vs. control)		0.3632	0.0533	0.0052

Bleomycin	Control	0.5 *μ*g/mL	1.0 *μ*g/mL	2.0 *μ*g/mL
Mean (Av. of 3 means)	16.75	20.00	24.97	37.22
Stnd. Dev., *n* = 3	1.331	4.521	2.146	5.784
Stand. error of mean	0.9415	3.197	1.518	4.090
*p* value (vs. control)		0.1925	0.0050	0.0041

EMS	Control	400 *μ*g/mL	800 *μ*g/mL	1600 *μ*g/mL
Mean (Av. of 3 means)	16.88	49.25	69.47	82.02
Stnd. Dev., *n* = 3	0.278	2.605	2.821	10.335
Stand. error of mean	0.1965	1.845	1.995	7.308
*p* value (vs. control)		<0.0001	<0.0001	0.0004

Statistical calculations are based on three sets of comet assays using aliquots of the same sets of treated samples.

**Table 2:** Statistical analysis of GC-MS/MS data (base lesions/10^6^ DNA bases).

	Control	0.5 *μ*g/mL	1.0 *μ*g/mL	2.0 *μ*g/mL
Bleomycin-FapyGua				
Mean (lesions/10^6^ bases)	0.8275	1.348	1.220	2.023
Stand. Dev., *n* = 4	0.1289	0.3316	0.2299	0.4723
Stnd. error of mean	0.06447	0.1658	0.1150	0.2362
*p* value (vs. control)		0.0133	0.0124	0.0014

Bleomycin-8-OH-Gua				
Mean (lesions/10^6^ bases)	0.9925	1.123	1.348	1.388
Stnd. Dev., *n* = 4	0.1658	0.2103	0.3432	0.4115
Stnd. error of mean	0.0829	0.1051	0.1716	0.2058
*p* value (vs. control)		0.1846	0.0559	0.0626

Bleomycin-5-OH-MeUra				
Mean (lesions/10^6^ bases)	0.5875	0.7400	0.8875	1.275
Stnd. Dev., *n* = 4	0.3567	0.02944	0.2971	0.3937
Stnd. error of mean	0.1783	0.01472	0.1486	0.1968
*p* value (vs. control)		0.2134	0.1219	0.0207

Statistical calculations are based on four sets of GC-MS/MS measurements using aliquots of the same sets of treated samples.

## Data Availability

Supporting data for the results of this report is available in the provided supplementary materials.

## Supplementary Materials

Supplementary Materials Figure S1: complete GC-MS/MS data comparing the induction of the DNA lesions by etoposide, bleomycin, and EMS. The first three lesions (8-OH Gua, FapyGua, and 5-OH-MeUra) show increasing trends with bleomycin (bleo) compared to the lack of any significant increases after treatment with etoposide (etop) and EMS (ems). The remaining lesions (FapyAde, 5-OH-5-MeHyd, 5-OH-Cyt, 5-OH-Ura, and 8-OH-Ade) did not show significant increases after treatment with etoposide, bleomycin, and EMS. Data is presented as the mean and standard deviation of 4 replicates.
