# Supplementary material for: Genotoxic Effects of Etoposide, Bleomycin, and Ethyl Methanesulfonate on Cultured CHO Cells: Analysis by GC-MS/MS and Comet Assay
Source: J Nucleic Acids. 2020 Jul 30;2020:8810105. doi: 10.1155/2020/8810105 (PMC7414336; doi:10.1155/2020/8810105)
Supplement: Supplementary Materials — Figure S1: complete GC-MS/MS data comparing the induction of the DNA lesions by etoposide, bleomycin, and EMS. The first three lesions (8-OH Gua, FapyGua, and 5-OH-MeUra) show increasing trends with bleomycin (bleo) compared to the lack of any significant increases after treatment with etoposide (etop) and EMS (ems). The remaining lesions (FapyAde, 5-OH-5-MeHyd, 5-OH-Cyt, 5-OH-Ura, and 8-OH-Ade) did not show significant increases after treatment with etoposide, bleomycin, and EMS. Data is presented as the mean and standard deviation of 4 replicates. [file 8810105.f1.pdf]

6/10/2020

Supplementary Materials

Genotoxic Effects of Etoposide, Bleomycin and Ethyl Methanesulfonate on Cultured CHO cells: Analysis by GC-MS/MS and Comet assay

Donald H. Atha*[,1], Erdem Coskun[2], Onur Erdem[3], Alessandro Tona[1], Vytas Reipa[1]

and Bryant C. Nelson[1]

[1] National Institute of Standards and Technology, Biosystems and Biomaterials Division, Materials Measurement Laboratory, Gaithersburg, MD 20899.

[2] University of Maryland, Institute for Bioscience and Biotechnology Research, Rockville, MD 20850.

[3] University of Health Sciences, Department of Toxicology, Gulhane Faculty of Pharmacy, Ankara, Turkey.

GC-MS/MS Analysis

We utilized gas chromatography-tandem mass spectrometry (GC-MS/MS) with isotope dilution using stable isotope-labelled analogues of analytes as internal standards to positively identify and accurately quantify the following DNA base lesions in genomic DNA: 8-OH-Gua, 5-OH-MeUra, 2,6-diamino-4-hydroxy-5-formamidopyrimidine (FapyGua), 4,6-diamino-5-formamidopyrimidine (FapyAde), 5-hydroxy-5-methylhydantoin (5-OH-5-MeHyd), 5-OH-Cyt, 5-OH-Ura and 8-OH-Ade. Aliquots of 8-OH-Gua-$^{15}N_5$, 5-OH-Me-Ura ^{13}C, $^{15}N_2$, FapyGua-^{13}C,$^{15}N_2$, FapyAde-^{13}C, $^{15}N_2$, $^{15}N_2$, 5-OH-5-MeHyd-^{13}C, 5-OH-Cyt-^{13}C, $^{15}N_2$, 5-OH-Ura-$^{13}C4$, $^{15}N_2$ and 8-OH Ade ^{13}C, $^{15}N_2$ were added as internal standards. The following mass transitions were used in mass spectrometric analysis: *m/z* 455 → *m/z* 440 and *m/z* 460 → *m/z* 445 for 8-OH-Gua and 8-OH-Gua-$^{15}N_5$, respectively; *m/z* 353 → *m/z* 343 and *m/z* 262 → *m/z* 347 for 5-OH-MeUra and 5-OH-MeUra ^{13}C, D, respectively; *m/z* 457 → *m/z* 368 and *m/z* 460 → *m/z* 371 for FapyGua and FapyGua-^{13}C, $^{15}N_2$, respectively; *m/z* 369 → *m/z* 354 and *m/z* 372 → *m/z* 357 for FapyAde and FapyAde- ^{13}C, $^{15}N_2$, respectively; *m/z* 346 → *m/z* 331 and *m/z* 349 → *m/z* 334 for 5-OH-5-MeHyd and 5-OH-5-MeHyd-^{13}C, $^{15}N_2$, respectively; *m/z* 343 → *m/z* 342 and *m/z* 346 → *m/z* 345 for 5-OH-Cyt and 5-OH-Cyt-^{13}C, $^{15}N_2$, respectively; *m/z* 344 → *m/z* 343 and *m/z* 350 → *m/z* 349 for 5-OH-Ura and 5-OH-Ura-$^{13}C4$, $^{15}N_2$, respectively; *m/z* 367 → *m/z* 352 and *m/z* 370 → *m/z* 355 for 8-OH Ade and 8-OH Ade ^{13}C, $^{15}N_2$ respectively. GC-MS/MS measurements were conducted as described previously (see main text Materials and Methods).

Figure S1: Complete GC-MS/MS data comparing the induction of the DNA lesions by etoposide, bleomycin and EMS. The first three lesions (8-OH Gua, Fapy Gua and 5-OH MeUra), show increasing trends with bleomycin (bleo) compared to the lack of any significant increases after treatment by etoposide (etop) and EMS (ems). The remaining lesions (FapyAde, 5-OH-5-MeHyd, 5-OH-Cyt, 5-OH-Ura, and 8-OH-Ade), did not show significant increases after treatment by etoposide, bleomycin and EMS. Data is presented as mean and standard deviation of 4 replicates.

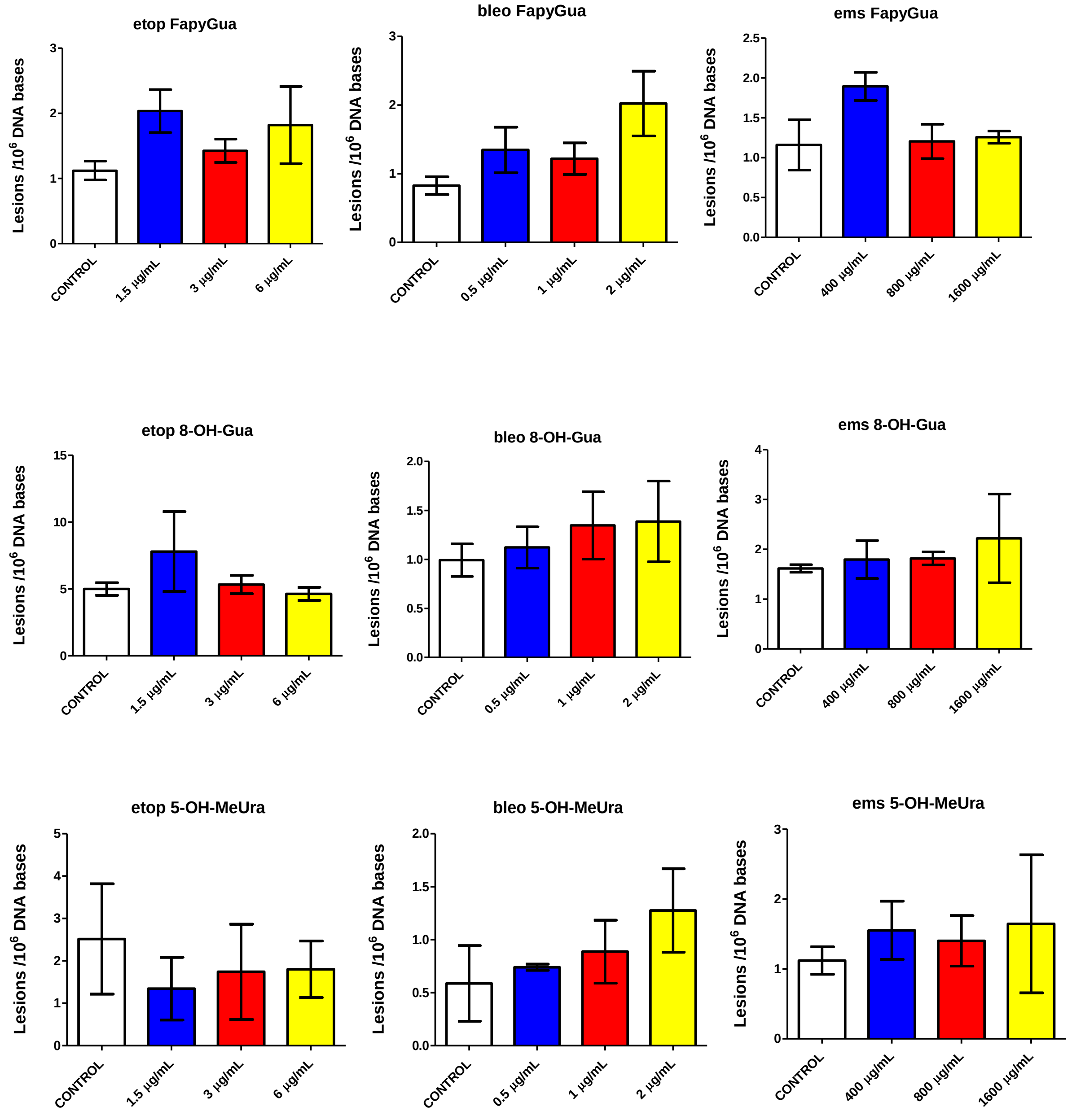

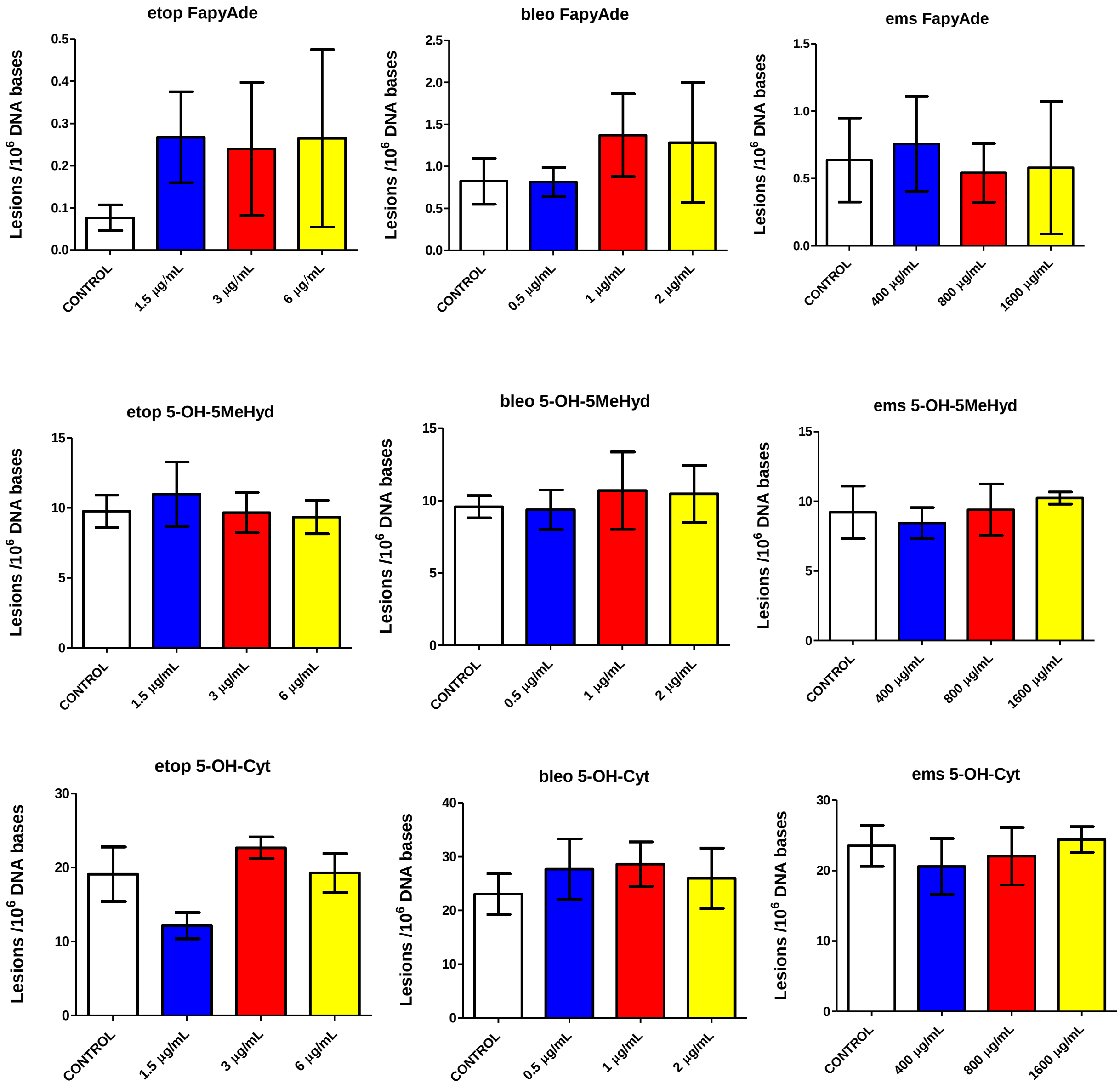
etop FapyAde
Lesions /10^6 DNA bases
0.0
0.1
0.2
0.3
0.4
0.5
CONTROL
1.5 µg/mL
3 µg/mL
6 µg/mL
bleo FapyAde
Lesions /10^6 DNA bases
0.0
0.5
1.0
1.5
2.0
2.5
CONTROL
0.5 µg/mL
1 µg/mL
2 µg/mL
ems FapyAde
Lesions /10^6 DNA bases
0.0
0.5
1.0
1.5
CONTROL
400 µg/mL
800 µg/mL
1600 µg/mL
etop 5-OH-5MeHyd
Lesions /10^6 DNA bases
0
5
10
15
CONTROL
1.5 µg/mL
3 µg/mL
6 µg/mL
bleo 5-OH-5MeHyd
Lesions /10^6 DNA bases
0
5
10
15
CONTROL
0.5 µg/mL
1 µg/mL
2 µg/mL
ems 5-OH-5MeHyd
Lesions /10^6 DNA bases
0
5
10
15
CONTROL
400 µg/mL
800 µg/mL
1600 µg/mL
etop 5-OH-Cyt
Lesions /10^6 DNA bases
0
10
20
30
CONTROL
1.5 µg/mL
3 µg/mL
6 µg/mL
bleo 5-OH-Cyt
Lesions /10^6 DNA bases
0
10
20
30
40
CONTROL
0.5 µg/mL
1 µg/mL
2 µg/mL
ems 5-OH-Cyt
Lesions /10^6 DNA bases
0
10
20
30
CONTROL
400 µg/mL
800 µg/mL
1600 µg/mL

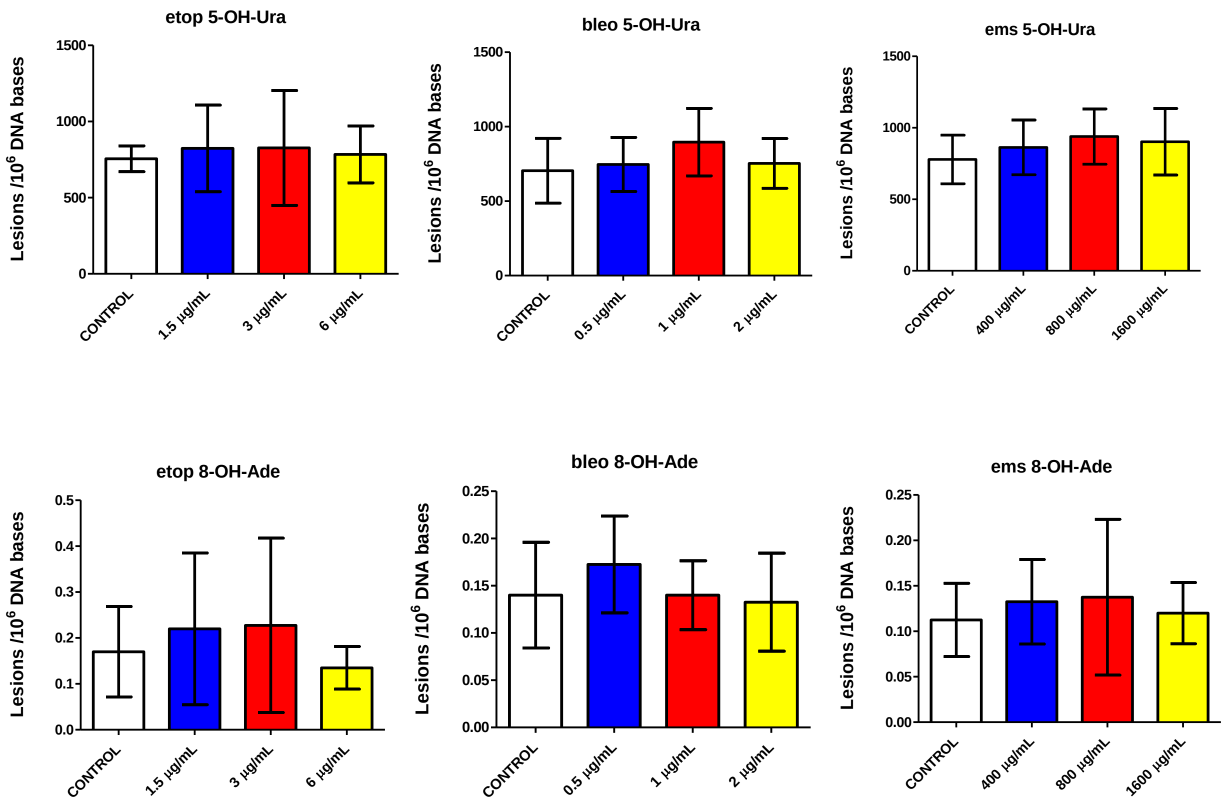
etop 5-OH-Ura
Lesions /10^6 DNA bases
0
500
1000
1500
CONTROL
1.5 µg/mL
3 µg/mL
6 µg/mL
bleo 5-OH-Ura
Lesions /10^6 DNA bases
0
500
1000
1500
CONTROL
0.5 µg/mL
1 µg/mL
2 µg/mL
ems 5-OH-Ura
Lesions /10^6 DNA bases
0
500
1000
1500
CONTROL
400 µg/mL
800 µg/mL
1600 µg/mL
etop 8-OH-Ade
Lesions /10^6 DNA bases
0.0
0.1
0.2
0.3
0.4
0.5
CONTROL
1.5 µg/mL
3 µg/mL
6 µg/mL
bleo 8-OH-Ade
Lesions /10^6 DNA bases
0.00
0.05
0.10
0.15
0.20
0.25
CONTROL
0.5 µg/mL
1 µg/mL
2 µg/mL
ems 8-OH-Ade
Lesions /10^6 DNA bases
0.00
0.05
0.10
0.15
0.20
0.25
CONTROL
400 µg/mL
800 µg/mL
1600 µg/mL
